# Current role of ultrasound in the diagnosis of hepatocellular carcinoma

**DOI:** 10.1007/s10396-020-01012-y

**Published:** 2020-03-13

**Authors:** Hironori Tanaka

**Affiliations:** Department of Gastroenterology and Hepatology, Takarazuka Municipal Hospital, 4-5-1 Kohama, Takarazuka, Hyogo Japan

**Keywords:** Hepatocellular carcinoma, Ultrasound, Contrast-enhanced ultrasound, Sonazoid, Fusion

## Abstract

Ultrasonography (US) is a major, sustainable hepatocellular carcinoma (HCC) surveillance method as it provides inexpensive, real-time, and noninvasive detection. Since US findings are based on pathological features, knowledge of pathological features is essential for delivering a correct US diagnosis. Recent advances in US equipment have made it possible to provide more information, such as malignancy potential and accurate localization diagnosis of HCC. Evaluation of malignancy potential is important to determine the treatment strategy, especially for small HCC. Diagnosis of blood flow dynamics using color Doppler and contrast-enhanced US is one of the most definitive approaches for evaluating HCC malignancy potential. Recently, a new Doppler microvascular imaging technique, superb microvascular imaging, which can detect Doppler signals generated by low-velocity blood flow, was developed. A fusion imaging system, another innovative US technology, has already become an indispensable technology over the last few years not only for US-guided radiofrequency ablation but also for the detection of small, invisible HCC. This article reviews the evidence on the use of ultrasound and contrast-enhanced ultrasound with Sonazoid for the practical management of HCC.

## Introduction

Ultrasonography (US) is a simple and noninvasive real-time imaging method available worldwide. Thus, it is the most frequently used imaging tool for diagnosing liver diseases. US is important not only for surveillance but also characterization of hepatocellular carcinoma (HCC). Most patients with HCC show liver cirrhosis (LC) with poor liver function and a high recurrence rate. Thus, early detection of HCC, especially in patients with LC, is important for timely treatment, which minimizes damage and preserves liver function.

While both computed tomography (CT) and magnetic resonance imaging (MRI) require contrast agents to detect small HCCs in most cases, US can detect most HCCs even without contrast agents. The use of contrast agents for CT and MRI is restricted in elderly patients with declining renal function. In addition, US is not associated with radiation exposure concerns. As US involves repeated examinations over a long period, it is suitable for the surveillance of HCC. Of course, using contrast agents improves the sensitivity and specificity of US; therefore, this method is useful for evaluating the malignancy grade of HCC, which is important when determining a treatment plan. Moreover, many new technologies such as fusion have improved our ability to diagnose HCC.

This article reviews the evidence on use of US and contrast-enhanced US with Sonazoid (Sonazoid CEUS) for the diagnosis and practical management of HCC.

## B-mode findings

In abdominal US, both detection and characterization play a role in diagnosing focal liver lesions. B-mode findings of HCC are the basis of diagnostic ultrasound, and the differential diagnosis is based on tumor shape, border and contour, tumor margin, and intratumoral and posterior echo [1]. The Terminology and Diagnostic Criteria Committee (TDCC) of Japan Society of Ultrasonics in Medicine (JSUM) summarizes HCC features of B-mode findings in detail (as presented in Table 1). These findings are useful for the differentiation of focal liver lesions.

**Table 1:** B-mode findings

Subtype	Shape	Border/contour	Tumor margin	Intra-tumor	Posterior echo	Additional findings
Nodular type (≤ 2 cm)	Round, roundish	Moderately well-defined, smooth	Hypoechoic peripheral zone (infrequent)	Various echo levels (mosaic pattern is sometimes observed)	Unchanged–sometimes enhanced	Bright loop
Nodular type (> 2 cm)	Round, roundish	Moderately well-defined, smooth	Thin hypoechoic peripheral zone (halo)	Mosaic pattern, nodule in nodule.(varies depending on the size and degree of differentiation)	Enhanced	Lateral echo
Massive type	Irregular shape	Poorly-defined		Various echo levels		

In addition, it is also important to understand US characteristics of HCC. Patterns of internal echoes of HCCs vary (hyperechoic pattern 12–38%, hypoechoic pattern 23–54%, mosaic pattern 17–38%) [2–5] depending on the size of the tumor [6].

**Fig. 1:**
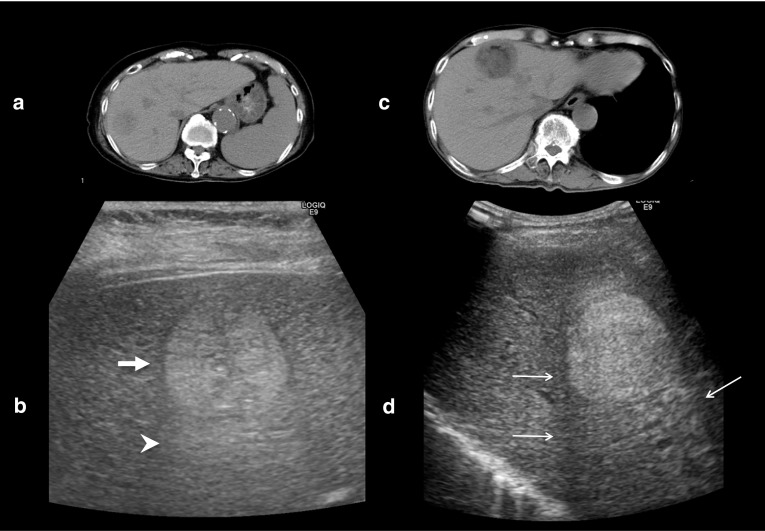
A case of newly developed hepatocellular carcinoma (HCC) (maximum diameter 26 mm) in Segment 7. Computed tomography (CT) shows a low attenuation area in Segment 7 (**a**). Conventional ultrasound shows a mosaic pattern nodule with posterior wall enhancement (arrowhead) and halo image (thick arrow) (**b**). A case of hepatitis C virus-related cirrhosis and a newly developed HCC in Segment 4 (**c**). Conventional ultrasound shows a high echo nodule with a typical lateral shadow (thin arrows) (**d**)

The internal echoes of HCCs smaller than 10 mm are almost hypoechoic (low level) or isoechoic, and the number of such low-level echoes increases with cell density. When tumor growth occurs as multistep hepatocarcinogenesis, fatty change is most frequently observed (36.4%) at a tumor diameter of 10–15 mm [7], and internal echoes of these HCCs are hyperechoic. When the diameter of an HCC reaches 20 mm or more, typical US patterns such as the “mosaic pattern,” “peripheral sonolucency (halo),” “lateral shadow,” and “posterior echo enhancement” can be recognized [8–11]. Findings of “mosaic pattern,” “posterior echo enhancement,” and “lateral shadow” show a higher accuracy (≥ 70%) and specificity (≥ 90%) in diagnosing HCC than metastatic liver cancer. With an increase in the size of the tumor, the frequency of observation of these US findings increases. However, these typical US findings are less frequently observed in smaller HCCs. The “halo sign” corresponds to the thin fibrous capsule of the HCC [12–15] (Figs. 1a, b). Correspondence between the sonographic halo sign and a histological capsule has been reported to be 90.1% [16]. The “lateral shadow,” which is a linear US feature observed at the edge of a tumor, represents the refraction that occurs when ultrasound passes through spherical tissue and the surrounding tissue at different speeds (Figs. 1c, d). Posterior echo enhancement arises posterior to any lesion that attenuates sound less than the surrounding tissue; the intensity of the transmitted ultrasound beam is relatively preserved distal to the lesion [3]. However, posterior echo enhancement is not specific to HCC; this finding is also associated with hemangiomas and cystic lesions.

In addition, the macroscopic configuration is important for the prediction of recurrence and prognosis in patients with HCC. In the classification proposed by a research group from Japan, the macroscopic configuration of HCC is divided into five types: small nodular type with indistinct margins, simple nodular type, simple nodular type with extranodular growth, confluent multinodular type, and infiltrative type [17, 18]. The potential for malignancy tends to change in accordance with the progression of the macroscopic configuration [11, 18].

## Color/power Doppler

Color Doppler can help visualize blood flow signals inside and at the margin of a tumor, and the direction of blood flow can be determined on the basis of color. Color Doppler helps identify not only nutrient vessels but also volume of blood flow. When it is necessary to detect the blood flow orthogonal to the ultrasonic beam or to evaluate low flow velocity, power Doppler is effective. Previously, power Doppler could increase the detection sensitivity, but it could not detect the blood flow velocity or direction. Now, with advances in technology, power Doppler can also detect the direction of blood flow.

JSUM’s TDCC also summarizes the HCC features of Doppler findings [1] (as presented in Table 2). In most HCCs smaller than 2 cm, blood flow is low and appears as lines or dots inside or around the tumor. Typical color Doppler features of a small HCC include afferent continuous waveform signals, which reflect a feeding portal flow [19]. When the diameter of a tumor is 2 cm or more, the blood flow volume increases. Especially in a moderately differentiated HCC that has a capsule and shows expansive growth, basket-pattern blood flow is observed. This pattern represents a fine network of arterial vessels that surrounds the tumor nodule [11, 20, 21]. Typical color Doppler features of an advanced HCC (massive type) include afferent pulsatile waveform signals associated with intratumoral continuous waveform signals and efferent continuous waveform signals [20].

**Table 2:** Doppler findings

Subtype	Blood flow	Vascularity	Blood flow characteristics (wave, stationary wave)	Additional findings
Nodular type (≤ 2 cm)	Low	Linear or dot-like vascularity is seen inside and around the tumor in some cases	Steady, sometimes pulsating	Blood-flow signals cannot be seen in many cases
Nodular type (> 2 cm)	High	Basket pattern (vascular network from the periphery to the center)	Pulsating, sometimes steady	A–P shunts and tumor emboli are seen in some cases
Massive type	High	Irregular vascularity, basket pattern	Pulsating	When pulsating flow is observed in the portal vein, tumor emboli or A–P shunts are suspected

**Fig. 2:**
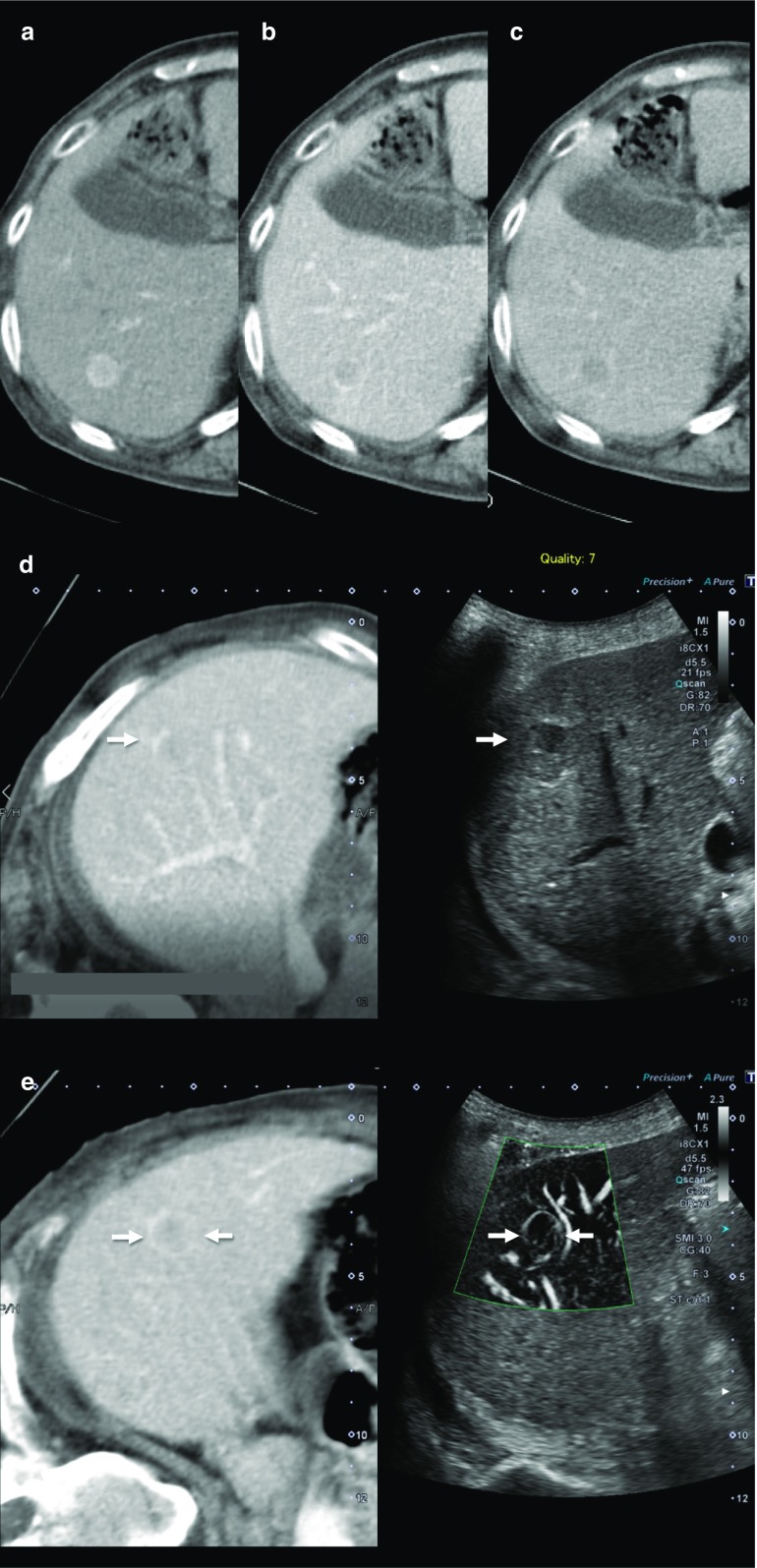
A case of recurrent HCC (maximum diameter 13 mm) in Segment 6. CT in the arterial phase shows hyper-enhancement (**a**). CT in the portal phase shows iso-enhancement (**b**). CT in the delayed phase shows hypo-enhancement (**c**). A hypoechoic tumor was found at the same site as the contrast-enhanced CT image in the portal phase using fusion imaging (**d**). Superb microvascular imaging (SMI) could produce very fine microvascular imaging with high resolution and a high frame rate (**e**)

Recently, a new Doppler microvascular imaging technique known as superb microvascular imaging (SMI; Canon Medical Systems, Otawara, Japan), which can distinguish Doppler signals generated by low-velocity blood flow from those generated by tissue movement, was developed. This technique can reduce motion artifacts and simultaneously provide a high level of sensitivity and imaging resolution at a high frame rate. As a result, ultrasound examiners using this technique can clearly observe low-velocity capillary blood flow without the need for a contrast agent. This increases confidence in the assessment of the nature of the tumors (Fig. 2) [22].

**Fig. 3:**
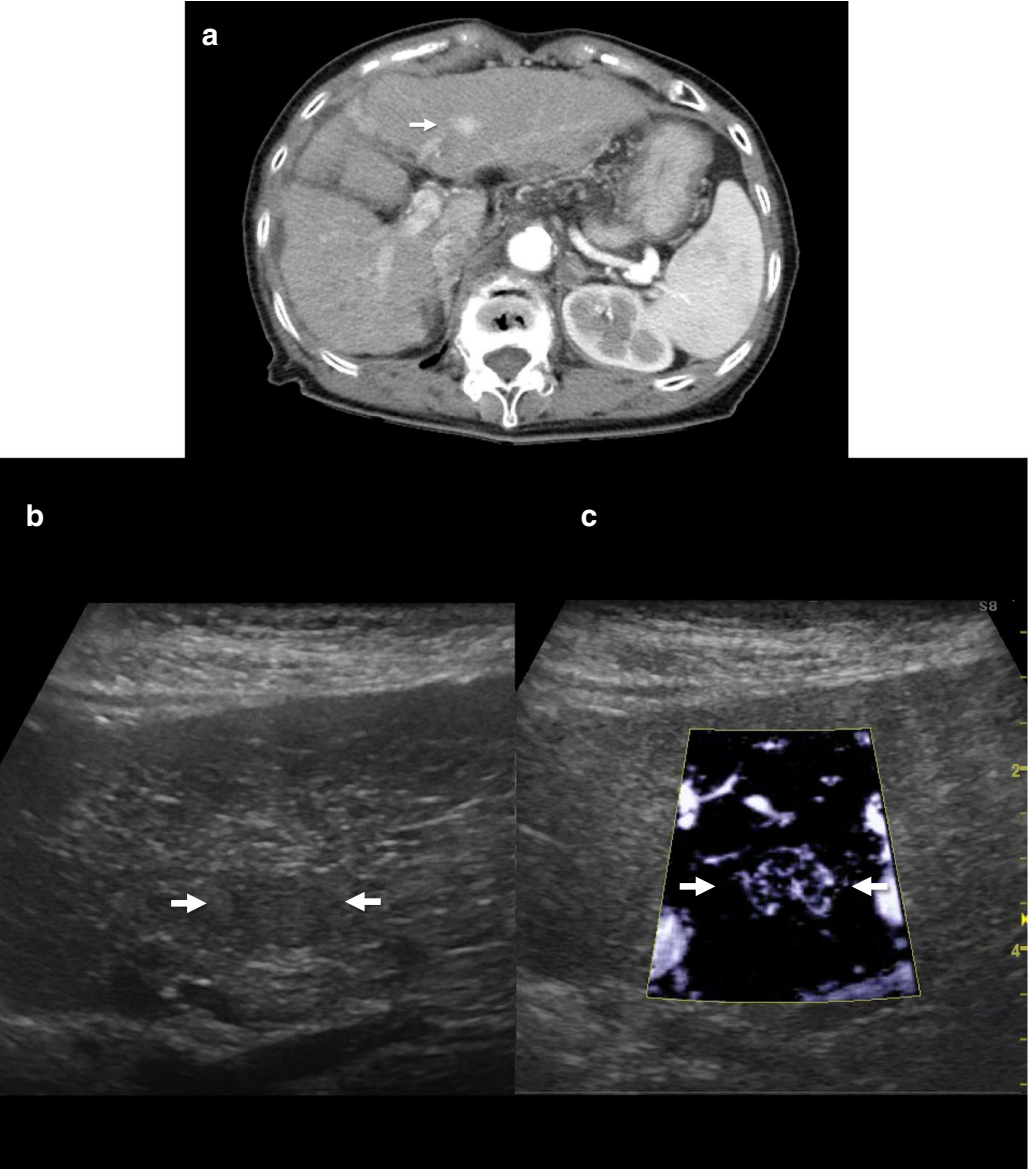
A case of newly developed HCC (maximum diameter 11 mm) in Segment 3. Contrast-enhanced CT in the arterial phase shows a high attenuation area (**a**). Although the tumor is tiny and hypoechoic (**b**), high-definition color (HDC) could produce very fine microvascular imaging with high resolution and a high frame rate (**c**)

Similar technology, such as high-definition color (HDC; GE Healthcare, Chalfont St. Giles, UK), has also become available and can display very small tumor vessels (Fig. 3). Although there are still few papers on the evaluation of HCC using these new Doppler microvascular imaging techniques, these are already being used in clinical practice in Japan. Thus, these blood flow display technologies may be used more in the future for HCC diagnosis using ultrasonography.

## Contrast-enhanced ultrasonography (contrast agent, techniques)

### Contrast agent and time phase

The first-generation agent Levovist (SH U 508A; Schering AG, Berlin, Germany) has been introduced not only in Japan but also in other countries [23]. However, the parenchyma-specific contrast yielded by Levovist is effective only when imaging is performed at high acoustic power using a high mechanical index (MI), and the effect is transient [24]. Therefore, it cannot be used for real-time examinations in the late phase, and visualization of the whole liver is limited to a single scan [25–27].

SonoVue (Bracco, Milan, Italy), widely available in Europe and China, enables continuous real-time imaging with a low MI [27–34]. SonoVue is minimally phagocytosed by reticuloendothelial cells (Kupffer cells) [35, 36]. Parenchyma-specific contrast images can be seen only 3–5 min after injection of SonoVue. Thus, its use is not approved in Japan [27].

In Japan, we can use only the second-generation ultrasound contrast agent Sonazoid (GE Healthcare, Amersham, UK); it is a lipid-stabilized suspension of perfluorobutane gas microbubbles, available since January 2007 [27]. Sonazoid is associated with a low incidence of side effects [37]. Because Sonazoid is metabolized in the lungs, it has no contraindication for patients with renal dysfunction or iodine allergy. Sonazoid CEUS is usually performed at a low MI. Because a low MI facilitates the prevention of microbubble destruction, obtaining perfusion images of hepatic lesions in the vascular phase using Sonazoid CEUS is easier. Sonazoid CEUS at a low MI also enables the user to scan the whole liver in the late phase, facilitating the detection of perfusion defect images of hepatic malignant lesions. With the employment of both the vascular and late phases, Sonazoid CEUS facilitates the characterization of liver tumors, histological grading of HCC lesions, and guided ablation of unresectable HCCs [27].

In humans, the reason for the enhancement of liver parenchyma and the non-enhancement of malignant tumors in the late phase using Sonazoid CEUS is not completely understood; however, it is thought to be a result of phagocytosis by reticuloendothelial (Kupffer) cells due to the adherence of microbubbles to the hepatic sinusoids and tumor vascular spaces or due to recirculation of Sonazoid microbubbles. Because malignant tumors contain few or no reticuloendothelial cells, they appear as perfusion defects in the late phase. The recommended dose of Sonazoid is 0.015 mL/kg. However, with an increase in the quantity of contrast agent, the ultrasound signal of the background liver may be enhanced simultaneously with that of the tumor at the time of imaging, which may make diagnosis difficult. This dose was determined on the basis of clinical research conducted at the initial introduction of this agent. Due to advances in US techniques, the image quality of Sonazoid CEUS is sufficient at doses lower than the recommended dose. Therefore, most authors injected a decreased dose of Sonazoid to evaluate the vascularity of liver lesions, especially HCCs [27, 38–41]. With regard to Sonazoid CEUS, time phases may be categorized into three subphases: arterial, portal, and post-vascular (Kupffer) phases [42]. The arterial phase is the period from the point of arrival of perflubutane microbubbles into the hepatic artery to the point when the portal vein and the hepatic artery intersect in the time-intensity curve (TIC). The portal phase is the period from the point when the hepatic artery and the portal vein intersect in the TIC to the point when the portal vein and the parenchyma intersect in the TIC. The post-vascular (Kupffer) phase (from 10 min after injection) starts when the parenchyma of the liver is enhanced after the disappearance of the contrast effect from the vasculature. JSUM’s TDCC also summarizes the HCC features of CEUS findings [1] (as presented in Table 3).

**Table 3:** Characterization by contrast-enhanced ultrasonography

Subtype	Vascular phase		Post-vascular phase	Additional findings
	Arterial [predominant]phase	Portal [predominant] phase	(Kupffer phase)	
Nodular type (≤ 2 cm)	Although a contrast agent sometimes infiltrates tumor vessels, only a few are visualized	Iso-enhancing or hypo-enhancing compared with liver parenchyma	Iso-enhancing or slightly hypo-enhancing compared with liver parenchyma	No enhancement in the arterial [predominant] phase in some cases
Nodular type (> 2 cm)	Basket pattern, vessel proliferation, irregular inflow vessels, stronger enhancement than liver parenchyma	Hypo-enhancing compared with liver parenchyma Has some non-enhancing regions	Defects or incomplete contrast defects	Dot-like signals remain in the post-vascular phase in some cases
Massive type	Basket pattern, vessel proliferation, irregular inflow vessels, inhomogeneous, stronger enhancement than liver parenchyma	Hypo-enhancing compared with liver parenchyma Has some non-enhancing regions	Defects or incomplete contrast defects, irregular tumor margin	Enhancement of tumor thrombus in some cases

**Table 4:**
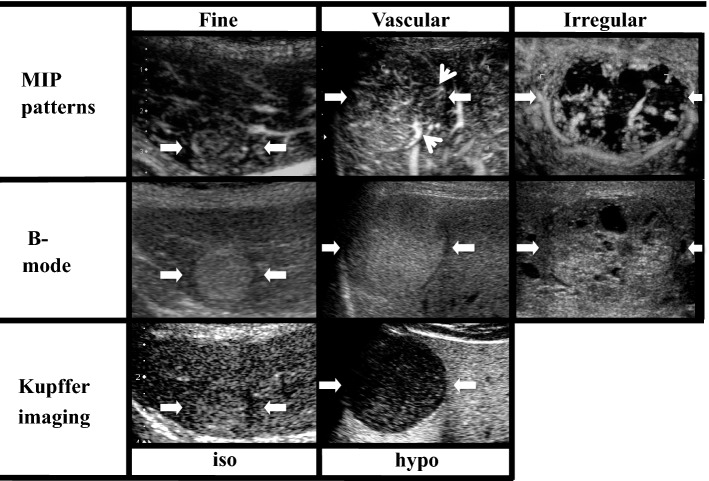
Classification of MIP and Kupffer imaging. The MIP pattern is classified as 1 of the following 3 patterns: (1) fine pattern: where tumor vessels were not clearly visualized and only fine vessels were visualized; (2) vascular pattern: where tumor vessels were visualized clearly; and (3) irregular pattern: where tumor vessels were thick and irregular. Small arrows in “vascular” category of MIP patterns show tumor vessels of vascular pattern. Kupffer imaging is classified as 1 of following 2 patterns: (1) iso-echoic pattern, (2) hypo-echoic pattern

### Defect reperfusion imaging

Differentiation of necrotic and viable areas is sometimes difficult in the post-vascular (Kupffer) phase as both appear as perfusion defects. To solve this problem, Kudo et al. re-injected Sonazoid into HCCs that had previously shown a perfusion defect in the post-vascular (Kupffer) phase [43]. They devised a method called defect reperfusion imaging, which confirms blood flow into the defects. Using this method, Hatanaka et al. reported that Sonazoid CEUS showed a higher sensitivity (95.4%) and accuracy (94.7%) in the diagnosis of hepatic malignancies than contrast-enhanced CT (sensitivity 85.2% and accuracy 82.3%) (both *P* < 0.005) [44].

### Contrast-enhanced low-MI tissue harmonic imaging

Contrast harmonic imaging (CHI) exploits the nonlinear oscillations of microbubbles in contrast agents that produce harmonic overtones of the original sound wave [45, 46]. Several contrast harmonic software applications have been developed for CEUS examination, with the most promising techniques being phase inversion (PI) and amplitude modulation (AM) [47, 48]. These techniques usually sacrifice spatial and time resolution to improve sensitivity and specificity of contrast signals.

**Fig. 4:**
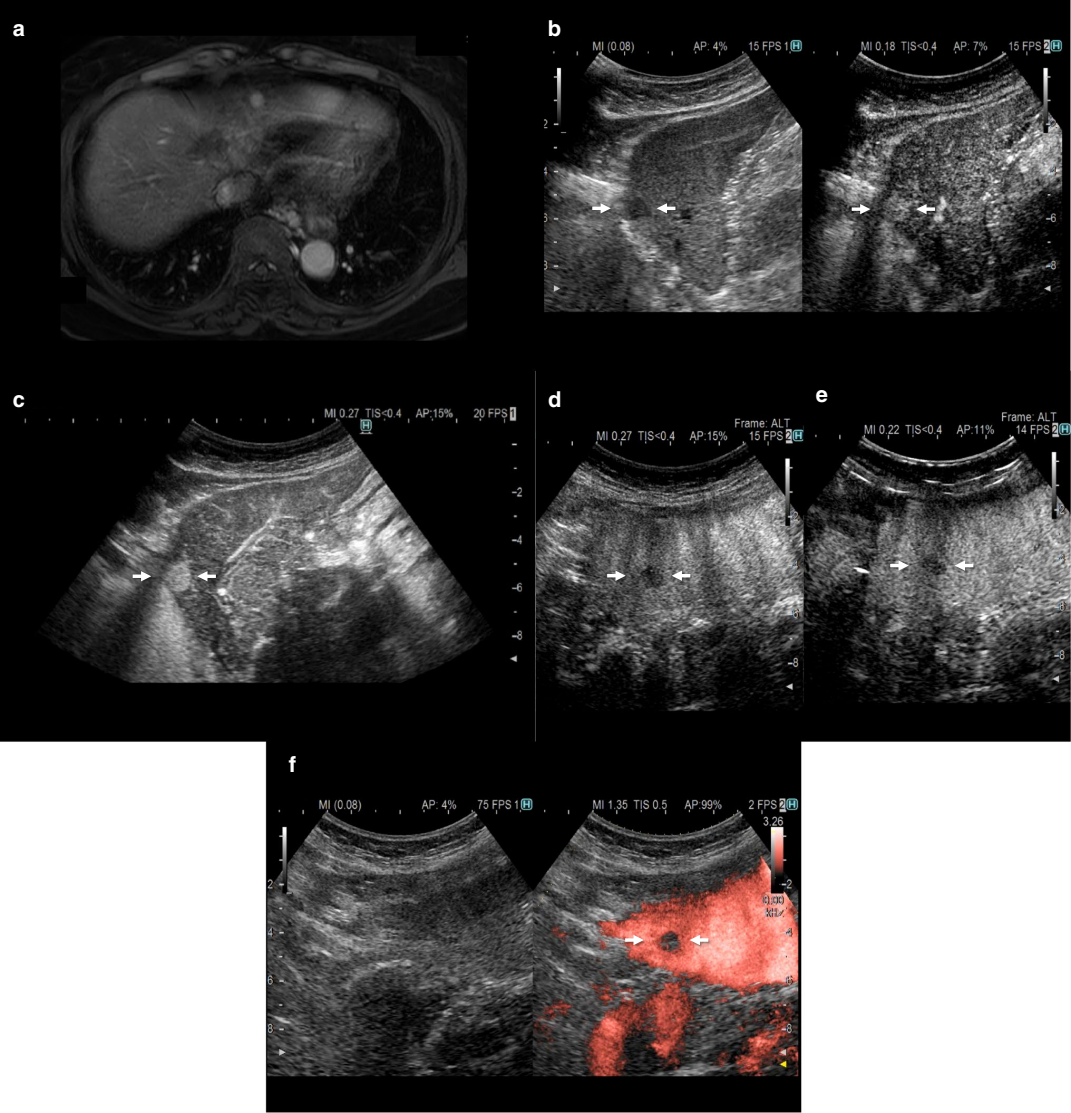
A case of newly developed HCC (maximum diameter 11 mm) in Segment 2. Hepatobiliary phase contrast-enhanced MRI with gadolinium ethoxybenzyl diethylenetriamine pentaacetic acid (Gd-EOB-DTPA) in the arterial phase shows a high-intensity area (**a**). This tumor is clearly enhanced with contrast-enhanced ultrasound with Sonazoid in the arterial phase using the phase inversion method (**b**). However, it is more clearly depicted by low mechanical index (MI) contrast-enhanced tissue harmonic imaging (THI) because of its high spatial and time resolutions (**c**). In the post-vascular (Kupffer) phase, the tumor is in the hypoechoic area by amplitude modulation, which is the mode most sensitive to contrast-enhanced ultrasound (**d**). We could also delineate the tumor with the same sensitivity by low MI contrast-enhanced THI (**e**). In addition, high-MI intermittent Doppler imaging could most clearly depict the tumor in the post-vascular (Kupffer phase) (**f**). The arrows point to the location of the identified hypoechoic tumor on imaging

**Fig. 5:**
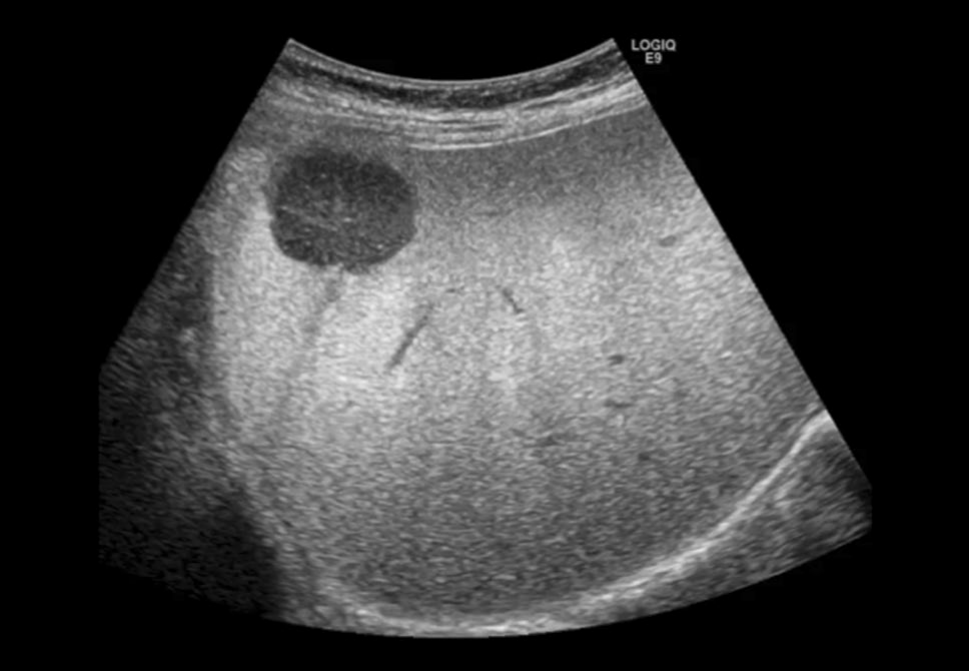
A case of metastatic liver cancer (maximum diameter 25 mm) in Segment 5. Low mechanical index (MI) contrast-enhanced THI clearly shows a low attenuation area

Tissue harmonic imaging (THI), which is the most commonly used conventional B-mode imaging modality, is a form of native harmonic imaging that provides a better signal-to-noise ratio and reduced side lobe artifacts than the other types [49–52]. As this THI technique is based on the PI technique, it must be used for CEUS. as well. Contrast low-MI THI, as a CEUS method, is a technique that simply lowers the MI value from more than 1.0 in general B-mode to less than 0.3. Therefore, contrast low-MI THI can offer an overlay view of conventional THI and contrast imaging [53]. This new CEUS method, also called low-MI harmonic imaging, is a technique that has been enabled by the recent evolution of ultrasound devices. It allows us to observe vessels in the vascular phase at high spatial and time resolutions using conventional B-mode US techniques such as compounding and time smoothing (Fig. 4c). Contrast low-MI THI using Sonazoid is also useful in the post-vascular (Kupffer) phase due to its high spatial and time resolutions (Fig. 5); however, the sensitivity is slightly lower than that of amplitude modulation, the most sensitive CEUS mode. Thus, low MI contrast THI is effective at providing detailed resolution, image quality, focal abnormality margin sharpness, and penetration for hepatic imaging.

### High-MI intermittent imaging

Numata et al. performed intermittent imaging at the rate of 2 frames/s in coded harmonic angio (CHA) mode at a high MI (0.7–1.2) to depict the features of tumor vascularity in the late phase (> 5 min). They called this method “high MI intermittent imaging.” When they used high-MI intermittent imaging to scan a tumor lesion, the Sonazoid microbubbles within and around the tumor may have been destroyed immediately, and the tumor vessels and tumor enhancements were possibly seen because of back flow into the tumor vessels and vascular spaces. In contrast, if the tumor became necrotic as a result of transcatheter arterial embolization, ablation therapy, or spontaneous necrosis, the lesions would exhibit neither tumor vessels nor enhancement when scanned using high-MI intermittent imaging. The advantages of this method include the acquisition of enough information from patients with multiple lesions. This method is regarded as an alternative to additional injection of contrast agents when tumor vascularity requires further evaluation, and it can be used when there is a lack of medical personnel or to save time [27, 38].

High-MI Doppler methods, such as advanced dynamic flow (ADF), may be used for evaluating HCC in the post-vascular (Kupffer) phase with the highest sensitivity and specificity, because microbubbles accumulated in the liver are destroyed by high-MI ultrasound exposure [54, 55]. The CEUS method can be performed only once as it destroys almost all the bubbles around the tumor on its first usage. Therefore, it is necessary to be careful not to destroy the bubbles accidentally. However, recent improvements have made it possible to perform these high-MI Doppler methods using only simple operations (Fig. 4f).

### Diagnosis of malignancy grade

Evaluation of malignancy potential is important for determining the treatment strategy, especially for small HCC. Although B-mode US can reflect the macroscopic morphology of HCC, only one study has investigated the relationship between B-mode ultrasonograms and the histological differentiation grade in patients with small HCC [56]. Moribana et al. classified small HCC into two groups ultrasonographically: type 1 (with halo) and type 2 (without halo). Type 2 was classified into three subgroups: type 2a, homogenous hyperechoic; type 2b, hypoechoic with a smooth margin; and type 2c, hypoechoic with an irregular or unclear margin. They showed that the malignancy potential of type 2a was the lowest and that of type 2c was the highest [56]. Their study is simple and important; however, many more studies have demonstrated the utility of CEUS in the assessment of malignancy.

## Evaluation in arterial phase

Details regarding the vascularity of an HCC are important, because blood supply and the grade of HCC malignancy are closely related. Hayashi et al. found a strong correlation between the intranodular arterial and portal supply evaluated by CT during hepatic arteriography and CT during arterial portography, and the malignancy grade of the hepatocellular nodules. In other words, the intranodular portal supply relative to the surrounding liver parenchyma observed by CT during arterial portography was decreased, whereas the intranodular arterial supply revealed by CT during hepatic arteriography was first decreased during the early stage of hepatocarcinogenesis, and then increased according to the malignancy grade of the nodules [57]. Thus, detection of hypervascularity in the arterial phase is important for assessing the malignancy grade of HCC. The sensitivity of CEUS in detecting the tumor vascularity of HCC nodules is equal to [58] or higher than [59] that of contrast-enhanced CT. Numata et al. demonstrated that the detection rate for hypervascularity of early HCC using CEUS (32.7%, 17/52) was significantly higher than that obtained using contrast-enhanced CT (21.2%, 11/52) (*P* < 0.005) [59]. Maruyama et al. also showed that CEUS detected hypervascularity in 26% (7/27) of the tumors characterized as non-hypervascular using contrast-enhanced CT [60].

## Evaluation in Kupffer phase

Superparamagnetic iron oxide (SPIO) (ferucarbotran [Resovist]; Bayer, Osaka, Japan) is a tissue-specific MRI contrast agent similar to Sonazoid; it is phagocytized by the liver Kupffer cells. SPIO-MRI imaging reflects the number of Kupffer cells and is useful for estimation of the histological grades of HCCs [61]. Inoue et al. compared the findings of Levovist-CEUS and SPIO-MRI and found a correlation between the findings of these methods in the post-vascular (Kupffer) phase. Post-vascular phase ratios (postcontrast echogenicity of the tumorous lesion/postcontrast echogenicity of the adjacent liver) declined as the nodules became less differentiated. The median values of the post-vascular phase ratios of dysplastic nodules, well-differentiated HCCs, and moderately and poorly differentiated HCCs were 1.01 (range 0.87–1.23), 0.75 (range 0.40–1.35), and 0.44 (range 0.07–0.61), respectively. The post-vascular phase ratios of dysplastic nodules and well-differentiated HCCs were significantly higher than those of moderately and poorly differentiated HCCs (both P < 0.001). However, the post-vascular phase ratios did not differ significantly between dysplastic nodules and well-differentiated HCCs (*P* = 0.02) [12].

Korenaga et al. compared the diagnostic ability of Sonazoid CEUS and SPIO-MRI in the post-vascular (Kupffer) phase using a quantitative parameter referred to as the Kupffer phase ratio (postcontrast echogenicity of tumorous lesion/postcontrast echogenicity of non-tumorous liver parenchyma).

These were nearly as significant as the post-vascular phase ratios used by Inoue et al., although there were differences in the numerical values depending on the differences in the echogenicity of their equipment. The mean values of the Kupffer phase ratios of well, moderately, and poorly differentiated HCCs were 0.836 ± 0.174 (range 0.595–1.280), 0.434 ± 0.125 (range 0.213–0.621), and 0.303 ± 0.091 (range 0.196–0.447), respectively. The Kupffer phase ratio using Sonazoid CEUS also decreased with a decrease in HCC differentiation [40]. In the Kupffer phase with Sonazoid CEUS, all moderately and poorly differentiated HCCs showed a hypoechoic pattern and were detected as perfusion defects; most (69.2%) of the well-differentiated HCCs showed an isoechoic pattern [40]. Sonazoid CEUS is also useful for estimating the histological grades of HCCs.

Gadolinium ethoxybenzyl diethylenetriamine pentaacetic acid (Gd-EOB-DTPA) has the properties of an extracellular and hepatocyte-specific contrast agent, and has recently played a leading role in the diagnosis of HCCs [62, 63]. Ohama et al. compared the histological enhancement patterns of the post-vascular (Kupffer) phase of Sonazoid CEUS and the hepatobiliary phase of Gd-EOB-DTPA-enhanced MRI, as well as uptake of Sonazoid and Gd-EOB-DTPA by HCC [64]. They evaluated the uptake of Sonazoid by TruAgent detection 30 min after administration of Sonazoid using Sonazoid enhancement indices [log (Doppler signals in the tumorous lesion) – log (mean of Doppler signals in the non-tumorous area]. TruAgent detection, the technique used to detect Doppler signals from the microbubbles of contrast agents, is based on the loss of correlation caused by the destruction of bubbles with high mechanical indices, ranging from 0.8 to 1.0. The median values of the Sonazoid enhancement indices were also decreased with a decrease in HCC histological differentiation: DNs 0 (range − 1.10 to 3.20); hypovascular well-differentiated HCCs 1.05 (range 5.65 to 3.60); hypervascular well-differentiated HCCs − 2.60 (range, − 0.82 to 0.40); moderately to poorly differentiated HCCs − 5.00 (range, − 8.60 to 2.20). In contrast, there was no difference in Gd-EOB-DTPA uptake across stages of HCC differentiation.

In addition, Kupffer images of Sonazoid CEUS can also be used to evaluate the gross HCC types that are closely related to malignancy potential. Hatanaka et al. classified HCC using Sonazoid CEUS into three types based on the macroscopic classification of the Liver Cancer Study Group of Japan [17, 65]: single nodular (SN) type, single nodular with extranodular growth (SNEG) type, and confluent multinodular (CMN) type. The ability of Sonazoid CEUS to correctly depict the gross HCC types was assessed. The sensitivity, specificity, and accuracy of Sonazoid CEUS were 96%, 80%, and 90%, respectively [66]. The diagnostic accuracy was 86.9% (53/61) for Sonazoid CEUS and 65.6% (40/61) for CECT [67].

Recently, a multicenter Japanese study showed that Kupffer phase images in Sonazoid CEUS could also predict hypervascularization of hypointense borderline lesions detected in the hepatobiliary phase of Gd-EOB-DTPA-enhanced MRI [HR: 3.684, 95% confidence interval: 1.798–7.546, *P* = 0.0004]. The cumulative incidence of hypervascularization of borderline lesions was 18%, 37%, and 43% at 1, 2, and 3 years, respectively [68].

### Maximum intensity projection

**Fig. 6:**
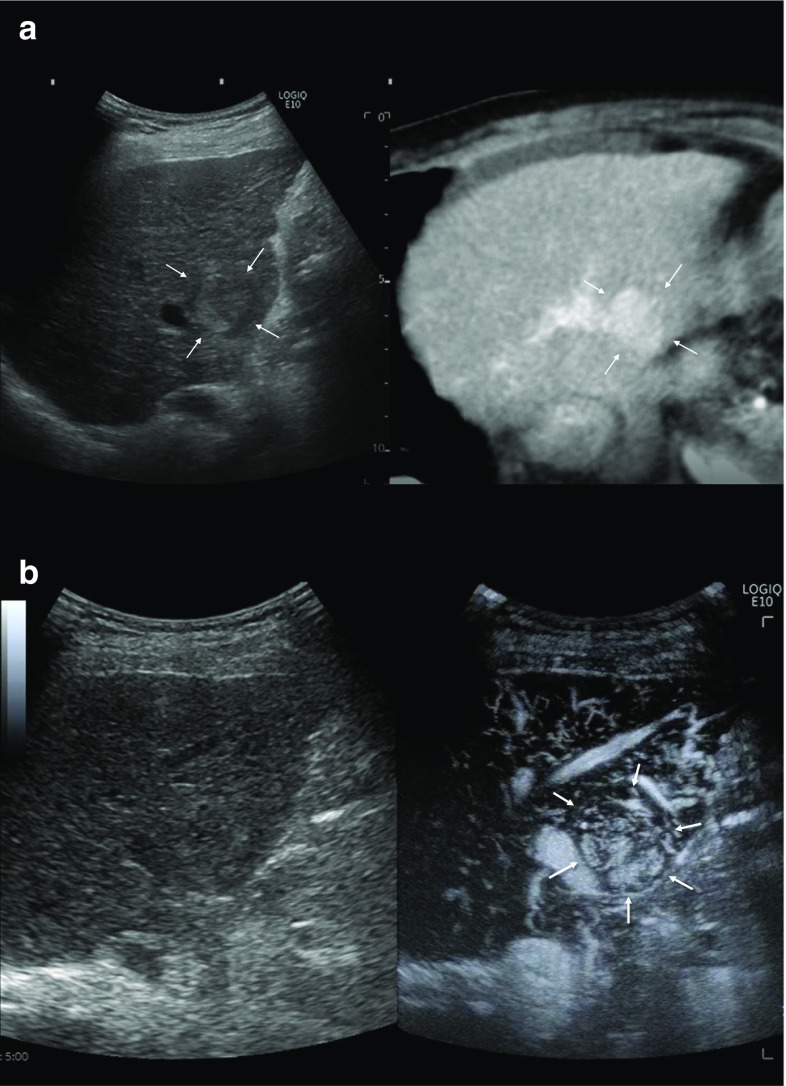
A case of newly developed HCC (maximum diameter 21 mm) in Segment 5. An isoechoic tumor was found at the same site as the contrast-enhanced CT image in the arterial phase using fusion imaging (**a**). Maximum intensity projection (MIP) can help visualize the fine vascular structure with the superior vessel continuity (**b**). The arrow points to the location of the identified isoechoic tumor on imaging

Micro-flow imaging (MFI), which is one of the CEUS methods for maximum intensity projection (MIP), is an accumulative imaging technique that reveals blood vessels after a flash with high-transmission power ultrasound exposure. In this modality, after the flash generated by the flash-replenishment sequence (FRS), the microbubbles in the scanning area are completely destroyed by the high energy output from the ultrasound beam. In succession, the area is re-perfused with microbubbles from the adjacent blood vessels, and the microbubbles are visible, because the imaging mode quickly shifts into a low-MI mode. Through maximum-holding image processing, the trace of the microbubbles in a temporal dimension can be clearly depicted, reflecting the vascularity in the scanning area [32]. HCC is known to undergo changes in vascular structure as it progresses. MIP can help visualize this fine vascular structure without an angiography of the liver, and the spatial resolution of MIP remains higher than that of SMI (Fig. 6).

Sugimoto et al. reported the feasibility of diagnosis of the histological differentiation of HCC using MIP with SonoVue CEUS. Briefly, the maximum-hold processing began immediately after the burst scan. The burst scan consisted of high-MI (1.3–1.6) scanning of five frames. Low-MI (0.16–0.30) scanning begun immediately after the MI burst scanning to visualize fresh microbubble contrast agent flowing into the scanning volume. The maximum intensity holding sequence started at the same time as the flash-replenishment low-MI imaging, which maintained the maximum brightness level on each pixel and displayed a persistent vision. They classified the image patterns as follows: normal, cotton, vascular, and dead-wood. In the normal pattern, the border between tumoral and non-tumoral regions is slightly indistinct, and the vascular architecture in the tumoral region is similar to that in the adjacent non-tumoral region. In the cotton pattern, the border between the tumoral and non-tumoral regions is distinct, but tumoral blood vessels are not clearly visualized, and the tumor appears pale as a whole as if it was stained. In the vascular pattern, tortuous and meandering tumoral blood vessels are clearly visualized, and the tumor is imaged as a whole. In the dead-wood pattern, tumoral blood vessels are clearly visualized, but they gradually taper off and are suddenly interrupted. HCCs showing normal and cotton patterns are classified as well differentiated; those showing vascular or dead-wood patterns are classified as moderately or poorly differentiated; the sensitivity, specificity, and accuracy of these assessments were found to be 85%, 92.7%, and 90%, respectively [69].

We also examined the clinical utility of the malignancy grading system to detect histologically advanced HCC using the MIP pattern. Our malignancy grading system was used to evaluate HCC histological grade with a combination of two key features of Sonazoid CEUS, i.e., Kupffer imaging and MIP pattern. Kupffer imaging was classified as an isoechoic or hypoechoic pattern. MIP patterns were evaluated using MFI and were classified as one of the following three patterns: fine (tumor vessels are not clearly visualized and only fine vessels are visualized), vascular (tumor vessels are clearly visualized), and irregular (tumor vessels are thick and irregular). An advantage of our MIP classification is the simplicity of its classification scheme. If some irregular vessels can be detected, the case is classified as an “irregular pattern”. Similarly, if some tumor vessels thicker than the surrounding fifth or sixth branch can be detected, the case is classified as a “vascular pattern”. Other cases were classified into a “fine pattern,” including cases in which the difference between the tumor vessels and surrounding hepatic parenchyma could not be detected. These MIP patterns of “fine,” “vascular,” and “irregular” are nearly equivalent to, respectively, Sugimoto’s MIP patterns of “normal or cotton,” “vascular,” and “dead-wood.” Simplification to three classifications has also made classification easier, especially for “normal or cotton” and “cotton or vascular” patterns, which are sometimes difficult to distinguish. Based on the combination of MIP patterns and Kupffer imaging, we were able to classify HCC into four grades: Grade 1 (iso-fine/vascular), Grade 2 (hypo-fine), Grade 3 (hypo-vascular), and Grade 4 (hypo-irregular) (Table 4). Reportedly, the distribution of moderately and poorly differentiated HCCs was as follows: Grade 1, 4% (1/24); Grade 2, 52% (15/29); Grade 3, 85% (44/52); and Grade 4, 100% (16/16). In this study, all patients with irregular patterns had either moderately or poorly differentiated HCC. In contrast, no patients with isoechoic patterns in Kupffer imaging had moderately or poorly differentiated HCC. Hence, combining Kupffer imaging with MIP patterns, we were able to histologically detect advanced HCC with a higher accuracy, and also predict portal vein invasion in 72 resected HCCs: Grade 1, 0% (0/4); Grade 2, 13% (1/8); Grade 3, 23% (11/48); and Grade 4, 67% (8/12) [70].

Takada et al. also reported the irregular MIP pattern, using Sonazoid CEUS, to be the most important independent risk factor for a poor outcome after successful radiofrequency ablation (RFA) in patients with early stage HCC (hazard ratio: 8.26, 95% confidence interval: 2.24–30.3, *P* = 0.002) [71].

As these study results demonstrate, MIP patterns are useful for estimating the malignancy potential of HCC. However, there remain some limitations to MIP analysis. First, the accuracy depends on the location, because it cannot be assessed without being clearly depicted. Second, there remains the possibility that key images cannot be detected, as only two-dimensional images of MIP patterns instead of the entire tumor are detectable. Therefore, these cases may be classified as being of a less malignant grade.

### Evaluation of treatment

Maruyama et al. summarized in detail the utility of Sonazoid CEUS for HCC treatment [41]. RFA is the most frequently used local treatment for HCC. Sonazoid CEUS could increase RFA applicability (from 21% [*n* = 95 cases] to 32% [*n* = 219 cases]) [72] and reduce the number of RFA sessions (from 1.49 ± 0.76 [historical control] to 1.33 ± 0.45) [73]. Using Sonazoid CEUS, Dohmen et al. demonstrated an increase in the non-local recurrence rate from 66.4 to 85.3% 2 years after RFA [74]. A recent study showed that Sonazoid CEUS performed 3 h after RFA could show the outline of the coagulated tumors in 78/87 patients (89.7%), and that the 5-year cumulative local recurrence rate was very low (2.3%) with a 5-year cumulative survival rate of 58.4% [75]. However, we should know two signs that indicate the risk of recurrence. The first sign is a linear-shaped positive enhancement, which was observed in the RFA-treated area in 33 lesions (18.4%) using Sonazoid CEUS; 17 of them were followed-up with no treatment, and three of these 17 (17.6%) showed local tumor progression that corresponded to linear enhancement [58]. The second sign is gradual intra-tumor enhancement in the pre-treatment early arterial phase, which indicates potential risk of distant recurrence after RFA [76].

Sonazoid CEUS is also useful for assessing therapeutic effects in transcatheter arterial chemoembolization (TACE), another important procedure for treating HCC. Xia et al. demonstrated that detection rates of residual tumors using Sonazoid CEUS 1 week after TACE were better than those using CT (58.1% vs. 39.5%, *P* < 0.001) [77]. A subsequent prospective study confirmed this result; it showed that the detection rate of residual HCC nodules using Sonazoid CEUS 1 day after TACE was significantly higher than that using contrast-enhanced CT 1 month after TACE (95.7% vs. 78.7%, *P* < 0.05) [78].

The first oral multi-targeted tyrosine kinase inhibitor, Sorafenib (Nexavar; Bayer Healthcare, Leverkusen, Germany), is recommended for unresectable advanced HCC. However, it is an expensive drug associated with certain adverse events. Therefore, evaluation of the early response is required. Sugimoto et al. demonstrated that tumor perfusion parameters were statistically significant on the basis of the area under the time-intensity curve (AUC) during wash-in on day 14, the most relevant factor indicating tumor response (*P* = 0.0016) [79]. Shiozawa et al. also examined the mean arrival time of Sonazoid using parametric imaging before and 2 weeks after treatment and suggested that an extended mean arrival time improves the median survival time (arrival time extend group 792 days, arrival time not extend group 403 days) [80].

## Cost-effectiveness

Surveillance of HCC using US improves the prognosis of patients [81–83], and it is a cost-effective method for evaluating patients with HCV-related cirrhosis [84, 85], even after a sustained virologic response to therapy [86]. The cost-effectiveness of CEUS for HCC surveillance in patients with LC was also reported [87]. Using the Markov model, we demonstrated that CEUS surveillance could cost effectively extend the expected survival time. Sensitivity analysis showed that the annual incidence of HCC and sensitivity of CEUS were two critical parameters. When the annual incidence of HCC was more than 2% and/or the CEUS sensitivity was more than 80%, the incremental cost-effectiveness ratio was less than the commonly accepted threshold of $US 50,000/QALY, indicating cost-effectiveness. However, economic conditions could vary over time, and the substantial effect is yet to be evaluated in each country.

## Innovation

### Fusion images

Fusion imaging is a novel technology that accurately combines real-time US images with real-time CT or MRI volume data and displays them on the same monitor, side by side. This means that a clinician can visualize both registered multiplanar reconstruction (MPR) images on the same monitor to make diagnostic or procedural decisions in real time. The fusion imaging system is composed of a position-sensing unit mounted on an ultrasound unit and an electromagnetic field transmitter. Some of the latest probe models are equipped with a position sensor.

US is the first choice for percutaneous interventional procedures as it provides real-time, noninvasive, repeatable, and non-radiation-based imaging. However, this process can be difficult if the ideal US scanning plane is different from that of the CT or MR image. Moreover, breathing and displacement and deformation of the abdominal structures due to pressure from the US probe can affect the process of mental registration. Lesions located deep in the most distal regions can be blurred and difficult to identify. In addition, US is affected by the presence of bone, gas, and fat. Real-time fusion imaging capitalizes on the strengths of all imaging modalities simultaneously. In addition, it allows for images acquired by means that are not affected by such issues, such as CT scanning and MR imaging, to be placed alongside or overlaid on the acquired images for better detection of lesions. US fusion imaging can also be associated with advanced US imaging techniques such as color/power Doppler, SMI, and CEUS for better localization and characterization of the lesions to be treated [88, 89].

Some hepatic tumors that can be visualized by CT or MR cannot be seen on US due to their small size, their location, or their echogenicity. In these cases, fusion imaging has been shown to make HCC nodules more conspicuous and to increase the feasibility of percutaneous RFA of HCCs not visible on conventional US [88, 90, 91] (Fig. 6). If HCCs remain non-visible after fusion imaging, anatomical landmarks surrounding the lesions can be used to guide correct needle placement [88]. Thus, with the use of fusion imaging, a larger population can benefit from US-guided ablation procedures instead of undergoing a CT-guided ablation or a major surgical operation, which are more invasive and expensive techniques. The average procedure duration for a US and CT/MRI fusion-guided liver biopsy was approximately half of that of a CT-guided liver biopsy (31.63 min vs 61.67 min, *P* = 0.003) [92]. Fusion imaging can also minimize false-positive lesion detection during US-guided RFA and consistently improve the detection of HCCs, especially when these are smaller than 20 mm [93]. The ability of fusion imaging to reduce false positives also applies to the evaluation of local tumor progression after RFA and TACE [94].

In recent years, next-generation microwaves systems, such as the Emprint Ablation System with Thermosphere Technology (Covidien, Boulder, CO), have been favored for their ability to consistently provide high intratumoral temperatures, fast ablation times, and large ablation volumes.[95]. Microwave ablation (MWA) is less susceptible to the heat sink effect because of its higher temperatures and shorter ablation times. Therefore, MWA needs to be used more carefully than RFA for its complications such as bile duct injuries, vascular damage, and visceral damage (colon, stomach, intestine, gallbladder, kidney, abdominal wall). To prevent such complications, a more accurate assessment of the predicted ablation area is necessary for MWA.

**Fig. 7:**
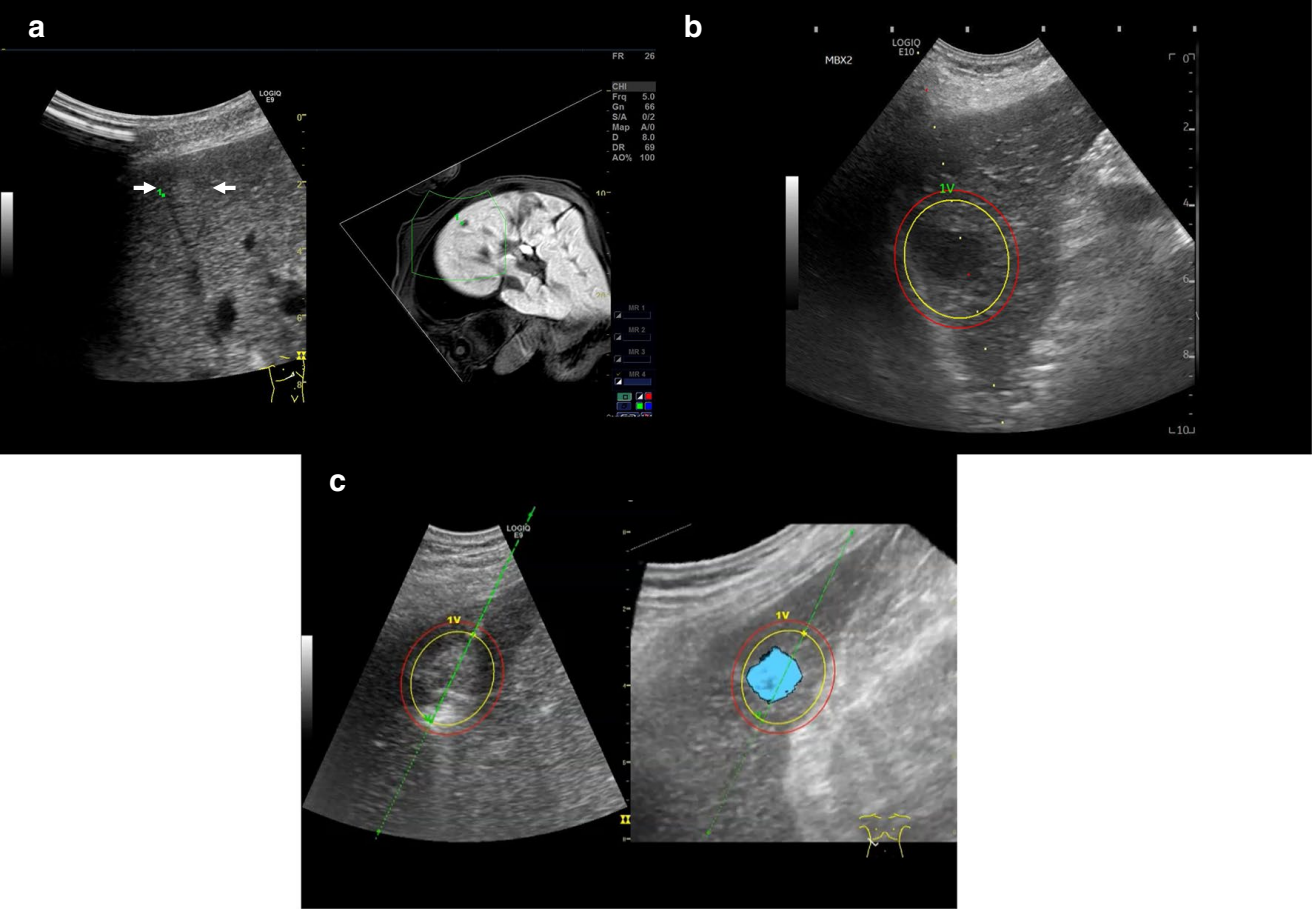
A case of recurrent HCC (maximum diameter 8 mm) in Segment 6. This tumor is detected by contrast-enhanced MRI with Gd-EOBDTPA only in the hepatobiliary phase. Because this tumor was very small, detection was also difficult with only B-mode ultrasound. However, using fusion technology, a high echo tumor could be visualized (**a**). Since the reconstructed magnetic resonance image can be enlarged and reduced freely, it is possible to search for lesions with ultrasound, while grasping the whole image with MRI (**a**). The 3D-GPS marker may easily change the size and shape of an ellipse. Using this function, operators may make more accurate simulations of how they ablate the tumor with sufficient margins in three dimensions (**b**). In addition, by combining a virtual needle-tracking system, a 3D-GPS marker may provide a more accurate prediction of the ablation area immediately prior to ablation. This needle-tracking system is able to virtually visualize the tip of the needle and the needle path on US. Thus, the combined use of this needle-tracking system and 3D-GPS marker at the tip of the RFA or MWA needle may successfully visually demonstrate the predicted ablation region (**c**)

Recently, we have also been able to use some new 3D display methods, such as 3D-GPS marker, which is one function of volume navigation (V Nav; GE Healthcare, Chalfont St. Giles, UK). This 3D-GPS marker may easily change the size and shape of an ellipse. Using this function, operators may make more accurate simulations of how they ablate the tumor with sufficient margins in three dimensions. By visually demonstrating the predicted ablation area in three dimensions, the operator can grasp the positional relationship between the ablation area and major blood vessels or other organs prior to ablation (Fig. 7b).

In addition, by combining a virtual needle-tracking system (VirtuTRAX; CIVCO Medical Solutions, Kalona, IA, USA) in which a magnetic sensor is attached to the distal tip of an ablation needle, a 3D-GPS marker may provide a more accurate prediction of the ablation area immediately prior to ablation [96]. This needle-tracking system is able to virtually visualize the tip of the needle and the needle path on US. Thus, the combined use of this needle-tracking system and 3D-GPS marker at the tip of the RFA or MWA needle may successfully visually demonstrate the predicted ablation region (Fig. 7c). A significantly bowed needle, however, may cause the virtual tract not to accurately match the actual needle. This problem may be solved using a more rigid needle such as a MWA needle.

It should be noted that the positional information derived by these 3D-GPS markers is not entirely consistent due to respiratory fluctuations, among other factors.

### Computer-aided diagnostic (CAD) systems

CEUS is useful for HCC diagnosis, but differential diagnosis is sometimes difficult. When microbubble destruction and variations in tumor behavior and liver enhancement occur, experienced sonographers (or radiologists) are required to characterize tumors reliably and accurately; however, the number of sonographers (or radiologists) experienced in performing and interpreting CEUS studies is limited, and interobserver agreement remains an issue. Computer-aided diagnostic (CAD) systems are a potential solution to these problems.

Generally, CAD systems extract features from the B-mode and/or CEUS videos and train machine-learning algorithms to associate these features with the known diagnoses to predict the diagnosis of unknown lesions. Sugimoto et al. proposed a method for classifying focal liver lesions into five classes (well-differentiated HCC, moderately differentiated HCC, poorly differentiated HCC, liver metastasis, and hepatic hemangioma) using four artificial neural networks (ANNs) for CAD analysis of focal liver lesions with Sonazoid CEUS. The accuracies of classifying hepatic hemangioma, HCC, and liver metastasis were 93.3%, 98.6%, and 84.8%, respectively. They also classified historical malignancy by CAD analysis using ANNs. The accuracies of classifying well-differentiated, moderately differentiated, and poorly differentiated HCC were 82.9%, 88%, and 100%, respectively [97].

Kondo et al. also proposed an automatic classification method based on machine learning for the interpretation of focal liver lesions on Sonazoid CEUS. This method yields spatial and temporal features in the arterial phase, portal phase, and Kupffer phase, as well as max-hold images. The lesions are classified as benign or malignant and again as benign, HCC, or metastatic liver tumor using support vector machines (SVM) with a combination of selected optimal features. Experimental results from 98 subjects indicated that benign and malignant classification showed 94.0% sensitivity, 87.1% specificity, and 91.8% accuracy, and that the accuracies of the benign, HCC, and metastatic liver tumor classifications were 84.4%, 87.7%, and 85.7%, respectively [98].

Reports on CAD are already available, and the practice is expected to be applied in clinical practice in the near future. There is no doubt that the introduction of deep learning will improve its accuracy. It will be necessary to develop a new diagnostic system to generate CAD-based results that enable experts to make reliable judgements.

## Conclusion

This comprehensive review clearly demonstrates the magnitude of the importance of US in the management of HCC. US examination of HCC has evolved significantly due to advances in equipment. First, B-mode has resulted in a marked improvement in image quality. In addition, using hepatobiliary phase contrast-enhanced MRI with Gd-EOB-DTPA and fusion technology, an innovative US-assisted system, even small lesions that were otherwise difficult to detect can be reliably analyzed. The visualized HCC can be evaluated for the very delicate hemodynamics of the tumor without the use of contrast agents by utilizing color Doppler, power Doppler, or SMI, which continue to evolve. Furthermore, when a contrast agent is used, a contrast pattern for each characteristic time phase can be evaluated, so that a more accurate diagnosis can be made. Although there remain certain parts that are difficult to visualize on ultrasonic examination, the high temporal and spatial resolution of US cannot be achieved using CT or MRI. Therefore, the usefulness, cost-effectiveness, and safety of US are indispensable. In the future, CAD will be incorporated into ultrasonic examinations. However, since tumor shape and blood flow evaluation remain unchanged, US will continue to play a central role in the diagnosis of HCC.

## References

[1] Terminology and Diagnostic Criteria Committee, Japan Society of Ultrasonics in Medicine (2014). Ultrasound diagnostic criteria for hepatic tumors. J Med Ultrason.

[2] Maringhini A, Cottone M, Sciarrino E (1988). Ultrasonography and alpha-fetoprotein in diagnosis of hepatocellular carcinoma in cirrhosis. Dig Dis Sci.

[3] Maturen KE, Wasnik AP, Bailey JE (2011). Posterior acoustic enhancement in hepatocellular carcinoma. J Ultrasound Med.

[4] Itai Y, Ohtomo K, Ohnishi S (1987). Ultrasonography of small hepatic tumors. Radiat Med.

[5] Choi BI, Kim CW, Han MC (1989). Sonographic characteristics of small hepatocellular carcinoma. Gastrointest Radiol.

[6] Nihei T, Ebara M, Ohto M (1992). Study of sonographic findings of small hepatocellular carcinoma based on its pathologic findings. Nihon Shokakibyo Gakkai Zasshi..

[7] Kutami R, Nakashima Y, Nakashima O (2000). Pathomorphologic study on the mechanism of fatty change in small hepatocellular carcinoma of humans. J Hepatol.

[8] Higashi T, Tobe K, Asano K (1988). Ultrasonographic characteristics of small hepatocellular carcinoma. Acta Med Okayama.

[9] Yoshida T, Matsue H, Okazaki N (1987). Ultrasonographic differentiation of hepatocellular carcinoma from metastatic liver cancer. J Clin Ultrasound.

[10] Inoue T, Kudo M, Watai R (2005). Differential diagnosis of nodular lesions in cirrhotic liver by post-vascular phase contrast-enhanced US with Levovist: comparison with superparamagnetic iron oxide magnetic resonance images. J Gastroenterol.

[11] Minami Y, Kudo M (2010). Hepatic malignancies: correlation between sonographic findings and pathological features. World J Radiol.

[12] Inoue T, Hyodo T, Korenaga K (2016). Kupffer phase image of Sonazoid-enhanced US is useful in predicting a hypervascularization of non-hypervascular hypointense hepatic lesions detected on Gd-EOB-DTPA-enhanced MRI: a multicenter retrospective study. J Gastroenterol.

[13] Tanaka S, Kitamura T, Imaoka S (1983). Hepatocellular carcinoma: sonographic and histologic correlation. AJR Am J Roentgenol.

[14] Wernecke K, Henke L, Vassallo P (1992). Pathologic explanation for hypoechoic halo seen on sonograms of malignant liver tumors: an in vitro correlative study. AJR Am J Roentgenol.

[15] Wernecke K, Vassallo P, Bick U (1992). The distinction between benign and malignant liver tumors on sonography: value of a hypoechoic halo. AJR Am J Roentgenol.

[16] Makuuchi M, Hasegawa H, Yamazaki S, et al. Ultrasonic characteristics of the small hepatocellular carcinoma. Ultrasound Med Biol. 1983;Suppl 2:489–91. 6100711

[17] Hui AM, Takayama T, Sano K (2000). Predictive value of gross classification of hepatocellular carcinoma on recurrence and survival after hepatectomy. J Hepatol.

[18] Shimada M, Rikimaru T, Hamatsu T (2001). The role of macroscopic classification in nodular-type hepatocellular carcinoma. Am J Surg..

[19] Tochio H, Tomita S, Kudo M (2002). The efferent blood flow of early hepatocellular carcinoma and borderline lesions: demonstration by color Doppler imaging. J Med Ultrason.

[20] Tochio H, Kudo M (2004). Afferent and efferent vessels of premalignant and overt hepatocellular carcinoma: observation by color Doppler imaging. Intervirology.

[21] Yen H-H (2018). Progress in the ultrasonographic microvascular imaging. J Med Ultrasound.

[22] Kudo M, Tochio H (2008). Intranodular blood supply correlates well with biological malignancy grade determined by tumor growth rate in pathologically proven hepatocellular carcinoma. Oncology.

[23] Marelli C (1999). Preliminary experience with NC100100, a new ultrasound contrast agent for intravenous injection. Eur Radiol.

[24] Albrecht T, Hoffmann CW, Schmitz SA (2001). Phase-inversion sonography during the liver-specific late phase of contrast enhancement: improved detection of liver metastases. AJR Am J Roentgenol.

[25] Blomley MJK, Sidhu PS, Cosgrove DO (2001). Do different types of liver lesions differ in their uptake of the microbubble contrast agent SH U 508A in the late liver phase?. Early Exp Radiol.

[26] Blomley MJK, Albrecht T, Cosgrove DO (1999). Improved imaging of liver metastases with stimulated acoustic emission in the late phase of enhancement with the US contrast agent SH U 508A: early experience. Radiology.

[27] Numata K, Luo W, Morimoto M (2010). Contrast enhanced ultrasound of hepatocellular carcinoma. World J Radiol.

[28] Quaia E, Calliada F, Bertolotto M (2004). Characterization of focal liver lesions with contrast-specific US modes and a sulfur hexafluoride-filled microbubble contrast agent: diagnostic performance and confidence. Radiology.

[29] Leen E, Ceccotti P, Kalogeropoulou C (2006). Prospective multicenter trial evaluating a novel method of characterizing focal liver lesions using contrast-enhanced sonography. Am J Roentgenol.

[30] Nicolau C, Vilana R, Catalá V (2006). Importance of evaluating all vascular phases on contrast-enhanced sonography in the differentiation of benign from malignant focal liver lesions. Am J Roentgenol.

[31] Leen E, Ceccotti P, Moug SJ (2006). Potential value of contrast-enhanced intraoperative ultrasonography during partial hepatectomy for metastases: an essential investigation before resection?. Ann Surg.

[32] Yang H, Liu G-J, Lu M-D (2007). Evaluation of the vascular architecture of hepatocellular carcinoma by micro flow imaging: pathologic correlation. J Ultrasound Med.

[33] Trillaud H, Bruel JM, Valette PJ (2009). Characterization of focal liver lesions with SonoVue-enhanced sonography: international multicenter-study in comparison to CT and MRI. World J Gastroenterol.

[34] Liu GJ, Xu HX, Xie XY (2009). Does the echogenicity of focal liver lesions on baseline gray-scale ultrasound interfere with the diagnostic performance of contrast-enhanced ultrasound?. Eur Radiol.

[35] Yanagisawa K, Moriyasu F, Miyahara T (2007). Phagocytosis of ultrasound contrast agent microbubbles by Kupffer cells. Ultrasound Med Biol.

[36] Lim AKP, Patel N, Eckersley RJ (2004). Evidence for spleen-specific uptake of a microbubble contrast agent: a quantitative study in healthy volunteers. Radiology.

[37] Moriyasu F, Itoh K (2009). Efficacy of perflubutane microbubble-enhanced ultrasound in the characterization and detection of focal liver lesions: phase 3 multicenter clinical trial. Am J Roentgenol.

[38] Numata K, Morimoto M, Ogura T (2008). Ablation therapy guided by contrast-enhanced sonography with Sonazoid for hepatocellular carcinoma lesions not detected by conventional sonography. J Ultrasound Med.

[39] Hatanaka K, Kudo M, Minami Y (2008). Differential diagnosis of hepatic tumors: value of contrast-enhanced harmonic sonography using the newly developed contrast agent. Sonazoid Intervirol.

[40] Korenaga K, Korenaga M, Furukawa M (2009). Usefulness of Sonazoid contrast-enhanced ultrasonography for hepatocellular carcinoma: comparison with pathological diagnosis and superparamagnetic iron oxide magnetic resonance images. J Gastroenterol.

[41] Maruyama H, Takahashi M, Ishibashi H (2009). Ultrasound-guided treatments under low acoustic power contrast harmonic imaging for hepatocellular carcinomas undetected by B-mode ultrasonography. Liver Int.

[42] Shunichi S, Hiroko I, Fuminori M (2009). Definition of contrast enhancement phases of the liver using a perfluoro-based microbubble agent, perflubutane microbubbles. Ultrasound Med Biol.

[43] Kudo M (2008). Hepatocellular carcinoma 2009 and beyond: from the surveillance to molecular targeted therapy. Oncology.

[44] Hatanaka K, Kudo M, Minami Y (2008). Sonazoid-enhanced ultrasonography for diagnosis of hepatic malignancies: comparison with contrast-enhanced CT. Oncology.

[45] Eckersley RJ, Chin CT, Burns PN (2005). Optimising phase and amplitude modulation schemes for imaging microbubble contrast agents at low acoustic power. Ultrasound Med Biol.

[46] Harvey CJ, Blomley MJ, Eckersley RJ (2000). Pulse-inversion mode imaging of liver specific microbubbles: improved detection of subcentimetre metastases. Lancet.

[47] Burns PN, Wilson SR, Simpson DH (2000). Pulse inversion imaging of liver blood flow: improved method for characterizing focal masses with microbubble contrast. Invest Radiol.

[48] Strobel D, Raeker S, Martus P (2003). Phase inversion harmonic imaging versus contrast-enhanced power Doppler sonography for the characterization of focal liver lesions. Int J Colorectal Dis.

[49] Chiou SY, Forsberg F, Needleman L (2007). Comparing differential tissue harmonic imaging with tissue harmonic and fundamental gray scale imaging of the liver. J Ultrasound Med.

[50] Shapiro RS, Wagreich J, Lao R (1998). Tissue harmonic imaging sonography: evaluation of image quality compared with conventional sonography. AJR Am J Roentgenol.

[51] Rosenthal SJ, Jones PH, Wetzel LH (2001). Phase inversion tissue harmonic sonographic imaging: a clinical utility study. AJR Am J Roentgenol.

[52] Sodhi KS, Sidhu R, Chawla Y (2005). Role of tissue harmonic imaging in focal hepatic lesions: comparison with conventional sonography. J Gastroenterol Hepatol.

[53] Kono M, Minami Y, Kudo M (2017). Contrast-enhanced tissue harmonic imaging versus phase inversion harmonic sonographic imaging for the delineation of hepatocellular carcinomas. Oncology.

[54] Shi WT, Forsberg F, Liu JB (2001). Blood flow estimation with harmonic Flash Echo Imaging. Ultrason Imaging.

[55] Suzuki S, Iijima H, Moriyasu F (2004). Differential diagnosis of hepatic nodules using delayed parenchymal phase imaging of Levovist contrast ultrasound: comparative study with SPIO–MRI. Hepatol Res.

[56] Moribata K, Tamai H, Shingaki N (2011). Assessment of malignant potential of small hypervascular hepatocellular carcinoma using B-mode ultrasonography. Hepatol Res.

[57] Hayashi M, Matsui O, Ueda K (1999). Correlation between the blood supply and grade of malignancy of hepatocellular nodules associated with liver cirrhosis: evaluation by CT during intraarterial injection of contrast medium. AJR Am J Roentgenol.

[58] Mandai M, Koda M, Matono T (2011). Assessment of hepatocellular carcinoma by contrast-enhanced ultrasound with perfluorobutane microbubbles: comparison with dynamic CT. Br J Radiol.

[59] Numata K, Fukuda H, Miwa H (2014). Contrast-enhanced ultrasonography findings using a perflubutane-based contrast agent in patients with early hepatocellular carcinoma. Eur J Radiol.

[60] Maruyama H, Takahashi M, Ishibashi H (2012). Contrast-enhanced ultrasound for characterisation of hepatic lesions appearing non-hypervascular on CT in chronic liver diseases. Br J Radiol.

[61] Imai Y, Murakami T, Yoshida S (2000). Superparamagnetic iron oxide-enhanced magnetic resonance images of hepatocellular carcinoma: correlation with histological grading. Hepatology.

[62] Ahn SS, Kim MJ, Lim JS (2010). Added value of gadoxetic acid-enhanced hepatobiliary phase MR imaging in the diagnosis of hepatocellular carcinoma. Radiology.

[63] Sano K, Ichikawa T, Motosugi U (2011). Imaging study of early hepatocellular carcinoma: usefulness of gadoxetic acid-enhanced MR imaging. Radiology.

[64] Ohama H, Imai Y, Nakashima O (2014). Images of Sonazoid-enhanced ultrasonography in multistep hepatocarcinogenesis: comparison with Gd-EOB-DTPA-enhanced MRI. J Gastroenterol.

[65] Shimada M, Rikimaru T, Hamatsu T (2001). The role of macroscopic classification in nodular-type hepatocellular carcinoma. Am J Surg.

[66] Hatanaka K, Chung H, Kudo M (2010). Usefulness of the post-vascular phase of contrast-enhanced ultrasonography with Sonazoid in the evaluation of gross types of hepatocellular carcinoma. Oncology.

[67] Hatanaka K, Minami Y, Kudo M (2014). The gross classification of hepatocellular carcinoma: usefulness of contrast-enhanced US. J Clin Ultrasound.

[68] Inoue T, Hyodo T, Korenaga K (2016). Kupffer phase image of Sonazoid-enhanced US is useful in predicting a hypervascularization of non-hypervascular hypointense hepatic lesions detected on Gd-EOB-DTPA-enhanced MRI: a multicenter retrospective study. J Gastroenterol.

[69] Sugimoto K, Moriyasu F, Kamiyama N (2008). Analysis of morphological vascular changes of hepatocellular carcinoma by microflow imaging using contrast-enhanced sonography. Hepatol Res.

[70] Tanaka H, Iijima H, Saito M (2014). New malignancy grading system for hepatocellular carcinoma using Sonazoid contrast enhanced ultrasonography. J Gastroenterol.

[71] Takada H, Tsuchiya K, Yasui Y (2016). Irregular vascular pattern by contrast-enhanced ultrasonography and high serum Lens culinaris agglutinin-reactive fraction of alpha-fetoprotein level predict poor outcome after successful radiofrequency ablation in patients with early-stage hepatocellular carcinoma. Cancer Med.

[72] Hiraoka A, Ichiryu M, Tazuya N (2010). Clinical translation in the treatment of hepatocellular carcinoma following the introduction of contrast-enhanced ultrasonography with Sonazoid. Oncol Lett.

[73] Masuzaki R, Shiina S, Tateishi R (2011). Utility of contrast-enhanced ultrasonography with Sonazoid in radiofrequency ablation for hepatocellular carcinoma. J Gastroenterol Hepatol.

[74] Dohmen T, Kataoka E, Yamada I (2012). Efficacy of contrast-enhanced ultrasonography in radiofrequency ablation for hepatocellular carcinoma. Intern Med.

[75] Nishigaki Y, Hayashi H, Tomita E (2015). Usefulness of contrast-enhanced ultrasonography using Sonazoid for the assessment of therapeutic response to percutaneous radiofrequency ablation for hepatocellular carcinoma. Hepatol Res.

[76] Maruyama H, Takahashi M, Shimada T (2013). Pretreatment microbubble-induced enhancement in hepatocellular carcinoma predicts intrahepatic distant recurrence after radiofrequency ablation. AJR Am J Roentgenol.

[77] Xia Y, Kudo M, Minami Y (2008). Response evaluation of transcatheter arterial chemoembolization in hepatocellular carcinomas: the usefulness of Sonazoid-enhanced harmonic sonography. Oncology.

[78] Takizawa K, Numata K, Morimoto M (2013). Use of contrast-enhanced ultrasonography with a perflubutane-based contrast agent performed one day after transarterial chemoembolization for the early assessment of residual viable hepatocellular carcinoma. Eur J Radiol.

[79] Sugimoto K, Moriyasu F, Saito K (2013). Hepatocellular carcinoma treated with sorafenib: early detection of treatment response and major adverse events by contrast-enhanced US. Liver Int.

[80] Shiozawa K, Watanabe M, Ikehara T (2017). Evaluation of sorafenib for advanced hepatocellular carcinoma with low a-fetoprotein in arrival time parametric imaging using contrast-enhanced ultrasonography. J Med Ultrason.

[81] Tanaka H, Nouso K, Kobashi H (2006). Surveillance of hepatocellular carcinoma in patients with hepatitis C virus infection may improve patient survival. Liver Int.

[82] Trevisani F, Cantarini MC, Labate AMM (2004). Surveillance for hepatocellular carcinoma in elderly Italian patients with cirrhosis: effects on cancer staging and patient survival. Am J Gastroenterol.

[83] Yu EW-R, Chie W-C, Chen TH-H (2004). Does screening or surveillance for primary hepatocellular carcinoma with ultrasonography improve the prognosis of patients?. Cancer J..

[84] Patel D, Terrault NA, Yao FY (2005). Cost-effectiveness of hepatocellular carcinoma surveillance in patients with hepatitis C virus-related cirrhosis. Clin Gastroenterol Hepatol.

[85] Thompson Coon J, Rogers G, Hewson P (2007). Surveillance of cirrhosis for hepatocellular carcinoma: systematic review and economic analysis. Health Technol Assess.

[86] Zangneh HF, Wong WWL, Sander B (2019). Cost effectiveness of hepatocellular carcinoma surveillance after a sustained virologic response to therapy in patients with hepatitis C virus infection and advanced fibrosis. Clin Gastroenterol Hepatol.

[87] Tanaka H, Iijima H, Nouso K (2012). Cost-effectiveness analysis on the surveillance for hepatocellular carcinoma in liver cirrhosis patients using contrast-enhanced ultrasonography. Hepatol Res.

[88] Lee MW (2014). Fusion imaging of real-time ultrasonography with CT or MRI for hepatic intervention. Ultrasonography.

[89] European Society of Radiology (ESR) (2019). Abdominal applications of ultrasound fusion imaging technique: liver, kidney, and pancreas. Insights Imaging.

[90] Song KD, Lee MW, Rhim H (2013). Fusion imaging-guided radiofrequency ablation for hepatocellular carcinomas not visible on conventional ultrasound. AJR Am J Roentgenol.

[91] Mauri G, Cova L, De Beni S (2015). Real-time US-CT/MRI image fusion for guidance of thermal ablation of liver tumors undetectable with US: results in 295 cases. Cardiovasc Intervent Radiol.

[92] Ahmed Y, Novak RD, Nakamoto D (2016). Is ultrasound fusion a reasonable replacement for computed tomography in guiding abdominal interventions?. J Ultrasound Med..

[93] Lee MW, Rhim H, Cha DI (2013). Planning US for percutaneous radiofrequency ablation of small hepatocellular carcinomas (1–3 cm): value of fusion imaging with conventional US and CT/MR images. J Vasc Interv Radiol.

[94] Min JH, Lee MW, Rhim H (2014). Local tumour progression after loco-regional therapy of hepatocellular carcinomas: value of fusion imaging-guided radiofrequency ablation. Clin Radiol.

[95] Simon CJ, Dupuy DE, Mayo-Smith WW (2005). Microwave ablation: principles and applications. Radiographics.

[96] Tomonari A, Tsuji K, Yamazaki H, Aoki H, Kang JH, Kodama Y (2013). Feasibility of the virtual needle tracking system for percutaneous radiofrequency ablation of hepatocellular carcinoma. Hepatol Res.

[97] Sugimoto K, Shiraishi J, Tanaka H (2016). Computer-aided diagnosis for estimating the malignancy grade of hepatocellular carcinoma using contrast-enhanced ultrasound: an ROC observer study. Liver Int.

[98] Kondo S, Takagi K, Nishida M (2017). Computer-aided diagnosis of focal liver lesions using contrast-enhanced ultrasonography with perflubutane microbubbles. IEEE Trans Med Imaging.

